# Defining Long-term Success after Anterior Augmentation Urethroplasty: 10-yr Patient-reported and Objective Outcomes According to the Novel Stricture-fecta Criteria

**DOI:** 10.1016/j.euros.2026.05.003

**Published:** 2026-05-20

**Authors:** Jakob Klemm, Robert J. Schulz, Navid Roessler, Max C. Wagner, Roland Dahlem, Margit Fisch, Malte W. Vetterlein

**Affiliations:** Department of Urology, University Medical Center Hamburg-Eppendorf, Hamburg, Germany

**Keywords:** Long-Term Outcome, Patient-Reported Outcome Measures, Quality of Life, Urethroplasty, Urethral Stricture

## Abstract

**Background and objective:**

There is a paucity of long-term data on patient-reported outcome measures (PROMs) after urethroplasty. We aimed to evaluate PROMs and retreatment-free survival more than 10 yr after anterior augmentation urethroplasty, using the recently established stricture-fecta criteria for standardized outcome reporting.

**Patients and methods:**

We retrospectively identified 97 men who underwent anterior augmentation urethroplasty between 2010 and 2013. Stricture characteristics were classified according to the Length, Segment, and Etiology system. Follow-up was performed via structured telephone interviews, and consenting patients completed a validated online survey at a single cross-sectional time point more than 10 yr after surgery. The survey included the Urethral Stricture Surgery (USS) PROM as well as instruments for urinary incontinence (ICIQ-UI SF), erectile function (IIEF-EF), and ejaculatory function (MSHQ-Ej). Retreatment-free survival was estimated using Kaplan-Meier analysis. The endpoints investigated met the trifecta criteria of standardized urethroplasty outcome reporting (stricture-fecta).

**Key findings and limitations:**

Median follow-up in 97 patients was 136 mo (95% confidence interval 133–138). Most strictures were >2 and ≤7 cm (66%) and bulbar (64%), predominantly iatrogenic (42%) or idiopathic (38%). Single-stage repair was performed in 90%. In total, 17 patients (18%) experienced recurrence, yielding 2-, 5-, and 10-yr retreatment-free survival rates of 90%, 86%, and 84%, respectively. Median USS PROM lower urinary tract symptoms score was 5/24, median ICIQ-UI SF 0/21, median IIEF-EF 19/30 (median modified IIEF-EF 24/30), and median MSHQ-Ej 28/35, indicating low urinary symptom burden and preserved sexual function. Mean EuroQol 5-Dimension (EQ-5D) index and EuroQol Visual Analogue Scale (EQ-VAS) were 0.936 and 78. Overall, 92% of patients reported being satisfied or very satisfied with their long-term outcomes. Limitations include the cross-sectional nature of long-term follow-up and potential nonresponse bias.

**Intervention:**

Augmentation urethroplasty.

**Conclusions and clinical implications:**

This first report of PROMs beyond 10 yr after anterior urethroplasty demonstrates durable retreatment-free survival, excellent long-term urinary and sexual function, and sustained patient satisfaction, providing a robust benchmark for current and emerging treatments.

## Take Home Message

Anterior augmentation urethroplasty delivers excellent long-term outcomes beyond 10 yr, including durable retreatment-free survival, preserved urinary and sexual function, and high patient satisfaction, establishing a robust benchmark for evaluating current and emerging treatments for anterior urethral stricture disease.

ADVANCING PRACTICE **What does this study add?**

This study provides the first comprehensive report of patient-reported outcomes more than 10 yr after anterior augmentation urethroplasty, assessed in accordance with the stricture-fecta framework. It demonstrates durable retreatment-free survival, preserved urinary continence and sexual function, and sustained patient satisfaction in a relatively young patient cohort. These findings establish a robust long-term, patient-centered benchmark for evaluating current and emerging treatments for anterior urethral stricture disease.

**Clinical Relevance**

Urethral stricture disease often affects young patients requiring durable treatment outcomes. In 97 men with a median follow-up of 136 mo, retreatment-free survival was 84% at 10 yr. Using validated PROMs fulfilling all stricture-fecta endpoints, low urinary symptom burden (USS PROM LUTS score 5/24), preserved erectile and ejaculatory function, and high overall health-related quality of life were demonstrated. Critically, 92% of patients reported being satisfied or very satisfied at long-term follow-up. These findings support the use of anterior augmentation urethroplasty as the gold standard and provide evidence-based data for patient counseling and comparative evaluation of minimally invasive alternatives.

**Patient Summary**

We studied men who had surgery to repair a narrowing of the urethra and asked them about their symptoms and quality of life more than 10 yr later. Most patients remained satisfied, with good urinary and sexual function and no need for further treatment. These results show that this type of surgery provides lasting benefits over the long term.

## 1 Introduction

Open urethroplasty remains the gold standard for the management of anterior urethral strictures, whereas endoscopic and device-based interventions—such as balloon dilation with or without drug coating, urethrotomy, and other minimally invasive approaches—may be considered in selected cases [1], [2]. Urethral stricture disease imposes a substantial burden on affected patients and often necessitates repeated medical interventions over time; however, uncertainty has long persisted regarding the optimal definition of treatment success [3].

In recent years, there has been a paradigm shift toward incorporating patient-reported outcome measures (PROMs) as key indicators of preoperative symptom burden and postoperative treatment success in urethral stricture disease [4], [5]. This shift is reflected in updated clinical guidelines, which now recommend the routine perioperative use of PROMs [1], [2]. In parallel, ongoing debate regarding the most clinically meaningful endpoints after urethral reconstruction [3], [6] has led an international collaborative to propose standardized urethral stricture trifecta criteria—the “stricture-fecta”—which define three nonnegotiable endpoints for the comprehensive assessment of outcomes following urethroplasty [7]. This framework aims to facilitate holistic, interpretable, and comparable outcome reporting across studies.

Despite these recommendations, long-term PROM data following urethroplasty that fulfill stricture-fecta criteria remain virtually nonexistent. This evidence gap is particularly relevant given the increasing use of alternative therapeutic options across diverse clinical settings, often beyond the scope of guideline recommendations and the available evidence [8]. Establishing a robust long-term evidence base is essential to rationalize treatment selection and ensure that the right therapy is delivered to the right patient with the right disease at the right time. Moreover, urethral stricture disease frequently affects relatively young patients, in whom long-term outcomes are of particular importance. Consequently, long-term data on the current standard of care are required to provide a benchmark against which emerging treatment strategies can be evaluated.

Therefore, we aimed to descriptively report long-term patient-reported and functional outcomes following anterior augmentation urethroplasty, with follow-up extending beyond 10 yr, in accordance with the stricture-fecta criteria. As no control group was included, causal inference was not intended; rather, our purpose was to establish a long-term benchmark to inform patient counseling and enable comparison with other treatment strategies.

## 2 Patients and methods

### 2.1 Study population and data extraction

This retrospective observational study was approved by the Ethics Committee of the Medical Council of Hamburg (PV4123) and conducted in accordance with local regulations (Hamburg Hospital Act, HmbKHG §12.1). We identified all male patients who underwent open augmentation urethroplasty for anterior urethral stricture disease between January 2010 and December 2013.

A comprehensive digital chart review was performed to extract data on patient demographics, stricture etiology, prior interventions, and surgical characteristics. Stricture features were further classified according to the Length, Segment, and Etiology classification of the Trauma and Urologic Reconstructive Network of Surgeons [9]. Long-term follow-up was conducted using structured telephone interviews, and PROMs were administered once at a single cross-sectional time point during long-term follow-up, more than 10 yr after the index surgery, using a secure web-based survey.

### 2.2 Study endpoints

Study endpoints comprised both objective and subjective outcomes and fulfilled all stricture-fecta criteria for standardized outcome reporting in urethral reconstruction, as recently defined through a modified Delphi consensus [7]. Objective outcomes consisted of functional success, defined as retreatment-free survival. Subjective outcomes were assessed using a set of validated PROMs, including the disease-specific Urethral Stricture Surgery Patient-Reported Outcome Measure (USS PROM) which incorporates the EuroQol 5-Dimension 5-Level questionnaire (EQ-5D-5L) [10], the International Consultation on Incontinence Questionnaire–Urinary Incontinence Short Form (ICIQ-UI SF) [11], the erectile function domain of the International Index of Erectile Function (IIEF-EF) [12], and the ejaculatory scale of the Male Sexual Health Questionnaire (MSHQ-Ej) [13].

### 2.3 Perioperative management and surgical procedure

All patients underwent preoperative evaluation in accordance with our institutional protocol, including a detailed medical history, physical examination, urinalysis, uroflowmetry, and combined retrograde urethrography and voiding cystourethrography.

All procedures were performed by two high-volume reconstructive urologists with extensive expertise in urethral surgery (RD, MF). To ensure a homogeneous study cohort, only patients undergoing augmentation urethroplasty were included; patients treated with anastomotic repairs were excluded.

Postoperatively, patients were typically discharged with an indwelling transurethral catheter and scheduled for follow-up after 21 d. At that visit, a voiding urethrogram was performed to confirm urethral patency and the absence of extravasation [14]. In cases of extravasation, the catheter was reinserted and imaging was repeated after 1 to 2 wk.

### 2.4 Statistical analyses

Baseline clinical characteristics were analyzed using descriptive statistics. Continuous variables are reported as medians with interquartile ranges (IQRs), except for EQ-5D-5L outcomes, which are presented as means with standard deviations (SDs) in accordance with the instrument’s user manual. Categorical variables are summarized as frequencies and proportions. Follow-up was defined as time from surgery to date of last contact. Patients without retreatment were censored at the date of last follow-up. Median follow-up time was estimated using the reverse Kaplan-Meier method, and retreatment-free survival was estimated using Kaplan-Meier analysis.

Potential nonresponse bias was addressed through two sensitivity analyses. First, baseline characteristics of PROM responders were compared with PROM nonresponders within the overall cohort using the Mann-Whitney U test, chi-square test, or Fisher’s exact test, as appropriate, to assess for systematic clinical differences that could introduce bias in both objective (RFS) and subjective (PROMs) outcome reporting. Second, retreatment rates were compared among patients successfully contacted by telephone, contrasting PROM responders and nonresponders to assess whether patients with less favorable surgical outcomes were less likely to complete the PROM questionnaires.

PROMs were evaluated according to their respective scoring manuals. Voiding symptoms were assessed using the lower urinary tract symptoms (LUTS) domain of the USS PROM, which comprises six items with a total score ranging from 0 to 24. Urinary continence was evaluated using the ICIQ-UI SF, consisting of three items with a total score ranging from 0 to 21; for both instruments, higher scores indicate greater symptom burden.

Erectile function was assessed using the IIEF-EF, which includes six items with a total score ranging from 1 to 30. Because the IIEF-EF does not adequately capture erectile function in men without recent sexual intercourse, an established modification proposed by Vickers et al was additionally applied, doubling the sum of the three nonintercourse items to provide an activity-independent erectile function score [15]. Ejaculatory function was evaluated using the MSHQ-Ej, which includes seven items and yields a total score ranging from 1 to 35.

General health status was assessed using the EQ-5D-5L, generating an index score between 0 and 1 across five dimensions (mobility, self-care, usual activities, pain/discomfort, and anxiety/depression), as well as a visual analogue scale (EQ-VAS), consistent with the original USS PROM methodology [10]. Treatment satisfaction was assessed using a single-item global satisfaction question from the USS PROM, rated on a four-point Likert scale ranging from very satisfied to very unsatisfied.

PROM scores are reported as medians with IQRs and means with SDs, and score distributions were visualized using violin plots. All statistical analyses were performed using Stata (StataCorp, Release 18; College Station, TX, USA).

Assistance in improving the English language and style was provided by ChatGPT (GPT-5, OpenAI) and was limited strictly to linguistic editing. The authors retained full responsibility for study design, data analysis, interpretation, and conclusions.

## 3 Results

### 3.1 Baseline characteristics

Of 494 patients who underwent open augmentation urethroplasty for anterior urethral stricture disease between 2010 and 2013 at our institution, 211 (43%) were reachable by telephone, and 97 (46%) of these completed the PROM questionnaires. Baseline characteristics of the PROM cohort are summarized in Table 1. The median age was 53 yr (interquartile range [IQR] 42–62), and the median body mass index was 26 (IQR 23–29). Seven patients (7.2%) had diabetes mellitus, and 18 (19%) had undergone prior open urethroplasty. Only three patients (3.1%) had received prior pelvic radiotherapy.

**Table 1:** Baseline characteristics of 97 patients undergoing anterior augmentation urethroplasty between 2010 and 2013 and completing PROM questionnaires at a long-term follow-up beyond 10 yr after the index procedure

Baseline characteristics	Patients included
No. of patients, *n* (%)	97
Age (yr), median (IQR)	53 (42–62)
BMI, median (IQR)	26 (23–29)
Diabetes, *n* (%)	7 (7.2)
Hypertension, *n* (%)	23 (24)
Coronary artery disease, *n* (%)	8 (8.2)
Prior pelvic radiotherapy, *n* (%)	3 (3.1)
Prior urethroplasty, *n* (%)	18 (19)
LSE classification, *n* (%)	
Length	
L1: ≤ 2 cm	16 (17)
L2: >2 cm and ≤7 cm	64 (66)
L3: >7 cm	17 (18)
Segment	
S1: Bulbar	62 (64)
S2: Penile	31 (32)
S3: Panurethral	4 (4.1)
Etiology	
E1: External trauma	6 (6.2)
E2: Idiopathic/unknown	37 (38)
E3: Iatrogenic	41 (42)
E4: Infectious/inflammatory	3 (3.1)
E5: Prior hypospadias repair	8 (8.2)
E6: Lichen sclerosus	2 (2.1)
Operative technique, *n* (%)	
One-stage	87 (90)
Staged	10 (10)
Follow-up (mo), median (95% confidence interval)*	136 (133–138)

BMI = body mass index; IQR = interquartile range; LSE = Length, Segment, Etiology; PROM = patient-reported outcome measures.

Percentages may not add up to 100%, as they are rounded.

* Follow-up duration was estimated using the reverse Kaplan-Meier method, applied to censored observations.

Most strictures measured >2 to 7 cm in length (L2: 66%), with the bulbar urethra representing the most common location (S1: 64%). The predominant stricture etiologies were iatrogenic (E3: 42%) and idiopathic (E2: 38%). Single-stage reconstruction was performed in 87 patients (90%), whereas 10 patients (10%) required multistage procedures.

A comparison of baseline characteristics between patients who completed the PROM questionnaires and those who did not is provided in Supplementary Table 1. Baseline characteristics were largely comparable between groups, with the exception of stricture etiology, suggesting that selection bias is unlikely to substantially affect results.

### 3.2 Objective outcomes

**Fig. 1:**
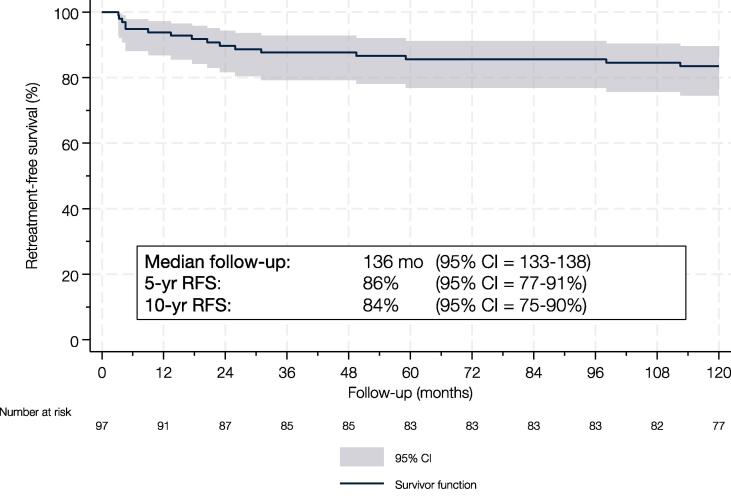
Kaplan-Meier curve depicting retreatment-free survival in 97 patients undergoing anterior urethroplasty between 2010 and 2013. CI = confidence interval.

The median follow-up among the 97 patients with available PROM data was 136 mo (95% confidence interval 133–138). During follow-up, 17 patients (18%) experienced stricture recurrence, resulting in retreatment-free survival rates of 90%, 86%, and 84% at 2, 5, and 10 yr, respectively (Fig. 1).

Management of recurrence included redo augmentation urethroplasty in eight patients, internal urethrotomy in seven, urethral dilation in one, and perineal urethrostomy in one.

In the overall cohort of 211 patients who could be reached by telephone for potential PROM assessment, retreatment had occurred more frequently among the 114 PROM nonresponders (34%) than among the 97 responders (18%; *p* = 0.008, Fisher’s exact test).

### 3.3 Patient-reported outcome measures

**Fig. 2:**
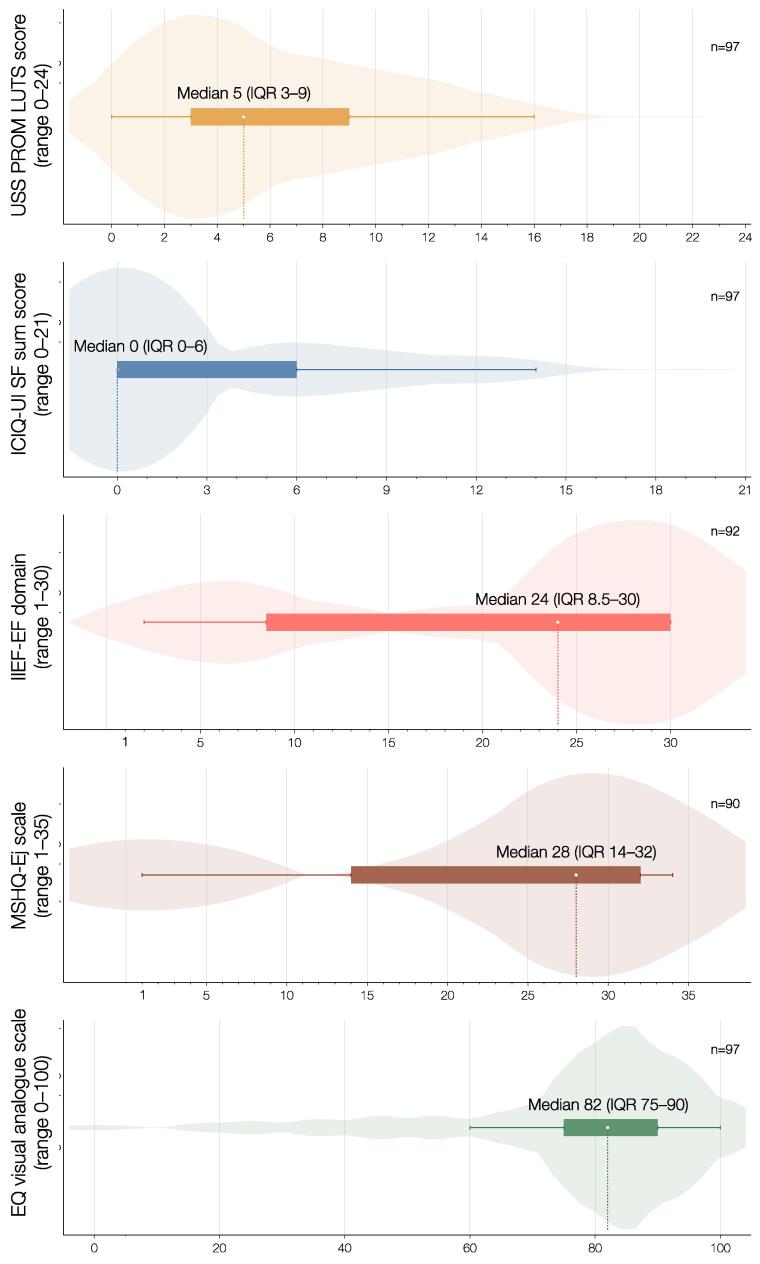
Violin plots illustrating the distribution of scores for validated patient-reported outcome measures in 97 patients undergoing anterior urethroplasty between 2010 and 2013. ICIQ-UI SF = International Consultation on Incontinence Questionnaire – Urinary Incontinence Short Form; IIEF-EF = International Index of Erectile Function, erectile function domain; MSHQ-Ej = Male Sexual Health Questionnaire, ejaculatory scale; USS PROM = Urethral Stricture Surgery Patient-Reported Outcomes Measure.

The median interval between surgery and PROM completion was 135 mo (IQR 129–143). PROM results are illustrated in Figure 2, and a detailed overview of response distributions is provided in Supplementary Table 2.

The median six-item LUTS score of the USS PROM was 5 (IQR 3–9) out of a maximum score of 24. The median ICIQ-UI SF score was 0 (IQR 0–6) out of a maximum score of 21, indicating a low burden of urinary symptoms.

The median IIEF-EF score was 19 (IQR 6.5–30). Using the activity-independent modification proposed by Vickers et al, the median modified IIEF-EF score was 24 (IQR 8.5–30), both out of a maximum score of 30. The median MSHQ-Ej score was 28 (IQR 14–32) out of a maximum score of 35, reflecting preserved sexual function.

The mean EQ-5D-5L index score was 0.936 (SD 0.132), and the mean EQ-VAS score was 78 (SD 19; range 0–100), indicating good overall health status.

Regarding treatment satisfaction, 57 patients (59%) reported being very satisfied, and 33 patients (34%) reported being satisfied with their urethral stricture treatment.

## 4 Discussion

To our knowledge, this is the first study to report patient-reported outcomes with long-term follow-up exceeding 10 yr after urethroplasty for anterior urethral strictures while adhering to the recently established stricture-fecta criteria for standardized outcome reporting [7]. To date, only one other study has reported outcomes beyond 10 yr of follow-up [16], underscoring the novelty and relevance of the present findings. With a median patient age of 53 yr, our cohort represents a relatively young population in whom long-term outcomes are particularly meaningful and reflective of the typical demographic undergoing urethral reconstruction.

In reconstructive urology, there has long been debate regarding which endpoints best define treatment success [6]. Traditional outcome measures—often summarized as “treatment success”—have suffered from inconsistent definitions, substantially limiting meaningful comparison across studies [6]. Consequently, clinical decision-making has historically relied more on individual experience than on robust, comparable outcome data. Against this backdrop, the incorporation of validated PROMs particularly in conjunction with long-term follow-up, provides critical insight into outcomes that are most relevant to patients. PROM-based evaluation improves patient selection, facilitates cross-study and cross-center comparisons, enables evidence-based counseling, and ultimately advances patient-centered care.

In this context, an international Delphi consensus recently proposed the stricture-fecta framework, defining freedom from retreatment, patient satisfaction, and the absence of clinically relevant impairment in urinary continence and sexual function as the three essential endpoints following urethral reconstruction [7]. The present study addresses a major evidence gap by reporting these outcomes more than a decade after surgery, thereby providing long-term validation of this framework.

The principal findings demonstrate that anterior augmentation urethroplasty is associated with excellent long-term outcomes, including sustained patient satisfaction more than 10 yr after surgery. Retreatment-free survival in our cohort is consistent with previously reported long-term success rates for anterior urethral strictures across different reconstructive techniques, which range from ∼66–88% at 10 yr [16], [17], [18], [19]. These data reinforce the durable effectiveness of urethroplasty as the standard of care.

Voiding outcomes, as assessed by the validated six-item LUTS domain of the USS PROM, were comparable to previously reported postoperative results from short- and mid-term studies across different languages and surgical approaches [10], [20], [21], [22]. Although earlier studies evaluated outcomes at shorter follow-up intervals, our findings suggest that low symptom burden is maintained well into the long term. Similarly, ICIQ-UI SF scores indicate a minimal burden of urinary incontinence, a finding that aligns with anatomical expectations for anterior urethral reconstruction and contrasts with the higher incontinence risk observed in posterior urethral disease.

Sexual function outcomes were likewise reassuring. Short-term erectile function after urethroplasty has been reported with variable results, although most studies suggest no lasting detrimental effect, with any transient decline typically resolving within 6 mo [23]. While it has been hypothesized that nontransecting techniques may better preserve vascular integrity and thus erectile function [24], robust supporting evidence remains limited [25]. In the present study, long-term IIEF-EF scores were comparable to previously reported short-term outcomes [26], [27], [28], [29], suggesting that anterior augmentation urethroplasty does not adversely affect erectile function in the long term. Nonetheless, future studies incorporating preoperative baseline assessments, multivariable adjustment for established risk factors, and longitudinal follow-up are needed to more definitively evaluate causal relationships.

Ejaculatory function similarly appears to be preserved in the long term. Although restoration of urethral patency may improve ejaculatory function, surgical injury to the bulbospongiosus muscle or perineal nerve can result in postoperative dysfunction [23]. The MSHQ-Ej scores observed in our cohort are consistent with previously reported short-term data following anterior urethroplasty [30], [31], supporting long-term preservation of ejaculatory function.

General health-related quality of life, as assessed by the EQ-5D index and EQ-VAS was comparable to that reported in the original USS PROM validation cohort [10], [20]. This finding indicates a relatively healthy patient population, in whom even modest functional impairments may disproportionately affect quality of life and treatment satisfaction—further emphasizing the importance of systematic PROM reporting.

Perhaps most importantly, our data demonstrate durable long-term patient satisfaction following anterior augmentation urethroplasty. These results provide a clinically meaningful benchmark derived from standardized, PROMs and objective retreatment-free survival, against which emerging and alternative treatment modalities can be compared.

Several limitations merit consideration, including the retrospective design, single-center setting, limited sample size, absence of preoperative baseline PROMs, and cross-sectional nature of long-term follow-up. PROMs were only collected at a single long-term time point and within-patient change could not be assessed. Nonetheless, this captures outcomes at a clinically meaningful interval when transient postoperative effects have resolved. Although we limited the cohort to patients undergoing augmentation urethroplasty to enhance homogeneity, variation in specific reconstructive techniques (e.g., graft type, graft placement, and surgical approach) may have influenced long-term outcomes. This heterogeneity reflects real-world reconstructive practice but may introduce variability in both functional and patient-reported results. Baseline characteristics were largely comparable between PROM responders and nonresponders, suggesting limited risk of systematic selection bias at inclusion. However, within the subgroup of patients who could be contacted by telephone, retreatment rates differed significantly between responders and nonresponders. Nonresponders more frequently had undergone retreatment, indicating a potential nonresponse bias. Specifically, patients with less favorable surgical outcomes may have been less likely to complete PROM assessments, which introduces the possibility that patient-reported outcomes are overestimated in the present study. Nonetheless, this study represents the first comprehensive report of long-term PROMs after urethroplasty and provides valuable evidence to inform patient counseling and future research. Prospective, multi-institutional studies with predefined PROM assessment time points are warranted to validate and expand upon these findings.

## 5 Conclusion

This study represents the first report of long-term patient-reported outcomes following anterior augmentation urethroplasty with follow-up extending beyond 10 yr. The results demonstrate that anterior augmentation urethroplasty provides durable recurrence-free survival, minimal long-term lower urinary tract and incontinence symptom burden, preserved sexual function, and high sustained patient satisfaction. These findings establish an important long-term benchmark derived from standardized, patient-centered outcomes, suitable for evaluating current and emerging treatments for anterior urethral stricture disease.

  ***Author contributions***: Malte W. Vetterlein had full access to all the data in the study and takes responsibility for the integrity of the data and the accuracy of the data analysis.

  *Study concept and design*: Klemm, Schulz, Fisch, Vetterlein.

*Acquisition of data*: Klemm, Schulz.

*Analysis and interpretation of data*: Klemm, Vetterlein.

*Drafting of the manuscript*: Klemm, Schulz, Vetterlein.

*Critical revision of the manuscript for important intellectual content*: Roessler, Wagner, Dahlem, Fisch.

*Statistical analysis*: Klemm, Vetterlein.

*Obtaining funding*: None.

*Administrative, technical, or material support*: None.

*Supervision*: None.

*Other* (specify): None.

  ***Financial disclosures:*** Malte W. Vetterlein certifies that all conflicts of interest, including specific financial interests and relationships and affiliations relevant to the subject matter or materials discussed in the manuscript (eg, employment/affiliation, grants or funding, consultancies, honoraria, stock ownership or options, expert testimony, royalties, or patents filed, received, or pending), are the following: None.

  ***Funding/Support and role of the sponsor*:** None.
