# Supplementary material for: Defining Long-term Success after Anterior Augmentation Urethroplasty: 10-yr Patient-reported and Objective Outcomes According to the Novel Stricture-fecta Criteria
Source: Eur Urol Open Sci. 2026 May 20;88:129–36. doi: 10.1016/j.euros.2026.05.003 (PMC13214351; doi:10.1016/j.euros.2026.05.003)
Supplement: Supplementary Data 1 [file mmc1.pdf]

Suppl. Table 1 – Baseline characteristics of 494 patients undergoing anterior augmentation urethroplasty between 2010 and 2013 stratified by PROM nonresponders versus PROM responders.

Baseline characteristics	**PROM nonresponders**	**PROM responders**	***p* value**
Number of patients, *n* (%)	397	97	
Age (yr), median (IQR)	55 (41-68)	53 (42-62)	0.1
BMI, median (IQR)	26 (24-29)	26 (23-29)	0.8
Diabetes, *n* (%)	31 (7.8)	7 (7.2)	0.8
Hypertension, *n* (%)	130 (33)	23 (24)	0.1
Coronary artery disease, *n* (%)	55 (14)	8 (8)	0.1
Prior pelvic radiotherapy, *n* (%)	29 (7.3)	3 (3.1)	0.1
Prior urethroplasty, *n* (%)	96 (24)	18 (19)	0.2
LSE classification, *n* (%)			
Length			0.3
L1: ≤ 2 cm	61 (15)	16 (17)	
L2: > 2 cm & ≤ 7 cm	288 (73)	64 (66)	
L3: > 7 cm	48 (12)	17 (18)	
Segment			0.5
S1: Bulbar	277 (70)	62 (64)	
S2: Penile	108 (27)	31 (32)	
S3: Panurethral	12 (3.0)	4 (4.1)	
Etiology			**0.01**
E1: External trauma	19 (4.8)	6 (6.2)	
E2: Idiopathic/unknown	86 (22)	37 (38)	
E3: Iatrogenic	235 (59)	41 (42)	
E4: Infectious/inflammatory	16 (4.0)	3 (3.1)	
E5: Prior hypospadias repair	38 (9.6)	8 (8.2)	
E6: Lichen sclerosus	3 (0.8)	2 (2.1)	
Operative technique, *n* (%)			0.6
One-stage	348 (88)	87 (90)	
Staged	49 (12)	10 (10)	

BMI = body mass index; IQR = interquartile range; LSE = Length, Segment, Etiology.
Percentages may not add up to 100%, as they are rounded.
