# Supplementary material for: Defining Long-term Success after Anterior Augmentation Urethroplasty: 10-yr Patient-reported and Objective Outcomes According to the Novel Stricture-fecta Criteria
Source: Eur Urol Open Sci. 2026 May 20;88:129–36. doi: 10.1016/j.euros.2026.05.003 (PMC13214351; doi:10.1016/j.euros.2026.05.003)
Supplement: Supplementary Data 2 [file mmc2.pdf]

Suppl. Table 2 – Comprehensive summary of patient-reported outcome measure (PROM) responses obtained at long-term follow-up extending beyond ten years after anterior urethroplasty.

**Urethral Stricture Surgery Patient-Reported Outcome Measure (USS PROM)**	*n* (%)
1. Is there a delay before you start to urinate?	
Never	40 (41)
Occasionally	34 (35)
Sometimes	18 (19)
Most of the time	4 (4.1)
All of the time	1 (1.0)
2. Would you say that the strength of your urinary stream is...	
Normal	33 (34)
Occasionally reduced	19 (20)
Sometimes reduced	23 (24)
Reduced most of the time	11 (11)
Reduced all of the time	11 (11)
3. Do you have to strain to continue urinating?	
Never	45 (46)
Occasionally	28 (29)
Sometimes	15 (15)
Most of the time	7 (7.2)
All of the time	2 (2.1)
4. Do you stop and start more than once while you urinate?	
Never	47 (48)
Occasionally	29 (30)
Sometimes	15 (15)
Most of the time	6 (6.2)
All of the time	0 (–)
5. How often do you feel your bladder has not emptied properly after you have urinated?	
Never	40 (41)
Occasionally	34 (35)
Sometimes	20 (21)
Most of the time	2 (2.1)
All of the time	1 (1.0)
6. How often have you had a slight wetting of your pants a few minutes after you had finished urinating and had dressed yourself?	
Never	39 (40)
Occasionally	27 (28)
Sometimes	15 (15)
Most of the time	13 (13)
All of the time	3 (3.1)
7. Overall, how much do your urinary symptoms interfere with your life?	
Not at all	53 (55)
A little	33 (34)
Somewhat	7 (7.2)
A lot	4 (4.1)

8. Please ring the number that corresponds with the strength of your urinary stream over the past month.	*n* (%)
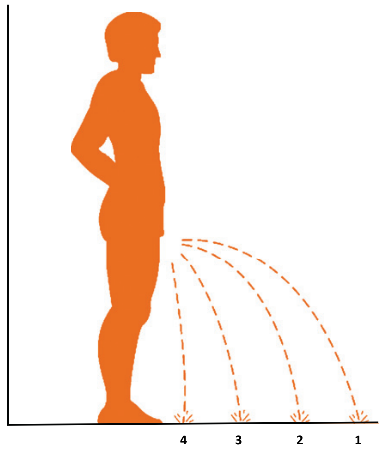	
1	12 (12)
2	41 (42)
3	31 (32)
4	13 (13)
9. Are you satisfied with the outcome of your operation?	
Yes, very satisfied	57 (59)
Yes, satisfied	33 (34)
No, unsatisfied	6 (6.2)
No, very unsatisfied	1 (1.0)

**EQ-5D-5L**<br>Under each heading, please tick the ONE box that best describes your health TODAY.	*n* (%)
**Mobility**	
I have no problems in walking about	84 (87)
I have slight problems in walking about	7 (7.2)
I have moderate problems in walking about	4 (4.1)
I have severe problems in walking about	0 (–)
I am unable to walk about	2 (2.1)
**Self-Care**	
I have no problems washing or dressing myself	94 (97)
I have slight problems washing or dressing myself	1 (1.0)
I have moderate problems washing or dressing myself	2 (2.1)
I have severe problems washing or dressing myself	0 (–)
I am unable to wash or dress myself	0 (–)
**Usual Activities**	
I have no problems doing my usual activities	87 (90)
I have slight problems doing my usual activities	7 (7.2)
I have moderate problems doing my usual activities	1 (1.0)
I have severe problems doing my usual activities	2 (2.1)
I am unable to do my usual activities	0 (–)
**Pain/Discomfort**	
I have no pain or discomfort	65 (68)
I have slight pain or discomfort	21 (22)
I have moderate pain or discomfort	4 (4.2)
I have severe pain or discomfort	6 (6.3)
I have extreme pain or discomfort	0 (–)
**Anxiety/Depression**	
I am not anxious or depressed	69 (73)
I am slightly anxious or depressed	17 (18)
I am moderately anxious or depressed	6 (6.4)
I am severely anxious or depressed	2 (2.1)
I am extremely anxious or depressed	0 (–)
**EQ VAS**; median (IQR)	82 (75–90)

International Consultation on Incontinence Questionnaire – Urinary Incontinence Short Form (ICIQ-UI SF)	*n* (%)
1. How often do you leak urine?	
Never	63 (65)
About once a week or less often	12 (12)
Two or three times a week	6 (6.2)
About once a day	3 (3.1)
Several times a day	11 (11)
All the time	2 (2.1)
2. We would like to know how much urine you think leaks. How much urine do you usually leak (whether you wear protection or not)?	
None	63 (65)
A small amount	31 (32)
A moderate amount	2 (2.1)
A large amount	1 (1.0)
3. Overall, how much does leaking urine interfere with your everyday life? Please ring a number between 0 (not at all) and 10 (a great deal)	
0 (not at all)	67 (69)
1	5 (5.2)
2	7 (7.2)
3	6 (6.2)
4	2 (2.1)
5	2 (2.1)
6	3 (3.1)
7	1 (1.0)
8	3 (3.1)
9	1 (1.0)
10 (a great deal)	0 (–)

Male Sexual Health Questionnaire (MSHQ) Ejaculation (Ej) Scale	*n* (%)
1. In the last month, how often have you been able to ejaculate when having sexual activity? (*n* = 94)	
All of the time	24 (26)
Most of the time	2 (2.1)
About half of the time	6 (6.4)
Less than half of the time	24 (26)
None of the time/Could not ejaculate	38 (40)
2. In the last month, when having sexual activity, how often did you feel that you took too long to ejaculate or "cum"? (*n* = 91)	
None of the time	29 (32)
Less than half of the time	17 (19)
About half of the time	15 (16)
Most of the time	6 (6.6)
All of the time	1 (1.1)
Could not ejaculate	23 (25)
3. In the last month, when having sexual activity, how often have you felt like you were ejaculating ("cumming"), but no fluid came out? (*n* = 90)	
None of the time	44 (49)
Less than half of the time	9 (10)
About half of the time	10 (11)
Most of the time	2 (2.2)
All of the time	5 (5.6)
Could not ejaculate	20 (22)
4. In the last month, how would you rate the strength or force of your ejaculation (*n* = 90)	
As strong as it always was	35 (38)
A little less strong than it used to be	11 (12)
Somewhat less strong than it used to be	10 (11)
Much less strong than it used to be	10 (11)
Very much less strong than it used to be	2 (2.2)
Could not ejaculate	22 (24)

5. In the last month, how would you rate the amount or volume of semen when you ejaculate? (*n* = 90)	
As much as it always was	38 (42)
A little less than it used to be	10 (11)
Somewhat less than it used to be	13 (14)
Much less than it used to be	6 (6.7)
Very much less than it used to be	1 (1.1)
Could not ejaculate	22 (24)
6. Compared to ONE month ago, would you say the physical pleasure you feel when you ejaculate has… (*n* = 91)	
Increased a lot	0 (–)
Increased moderately	1 (1.1)
Neither increased nor decreased	65 (71)
Decreased moderately	3 (3.3)
Decreased a lot	2 (2.2)
Could not ejaculate	20 (22)
7. In the last month, have you experienced any physical pain or discomfort when you ejaculated? Would you say you have… (*n* = 92)	
No pain at all	65 (71)
Slight amount of pain or discomfort	4 (4.4)
Moderate amount of pain or discomfort	1 (1.1)
Strong amount of pain or discomfort	0 (–)
Extreme amount of pain or discomfort	0 (–)
Could not ejaculate	22 (24)
8. In the last month, if you have had any ejaculation difficulties or have been unable to ejaculate, have you been bothered by this? (*n* = 72)	
Not at all bothered	49 (68)
A little bit bothered	10 (14)
Moderately bothered	4 (5.6)
Very bothered	6 (8.3)
Extremely bothered	3 (4.2)

**International Index of Erectile Function (IIEF) – Erectile Function Domain (EF)**<br>Over the past 4 weeks....	*n* (%)
1. How often were you able to get an erection during sexual activity?	
No sexual activity	18 (19)
Almost never or never	7 (7.2)
A few times (less than half the time)	5 (5.2)
Sometimes (about half the time)	11 (11)
Most times (more than half the time)	11 (11)
Almost always or always	45 (46)
2. When you had erections with sexual stimulation, how often were your erections hard enough for penetration? (*n* = 94)	
No sexual activity	18 (19)
Almost never or never	13 (14)
A few times (less than half the time)	3 (3.2)
Sometimes (about half the time)	9 (9.6)
Most times (more than half the time)	11 (12)
Almost always or always	40 (43)
3. When you attempted intercourse, how often were you able to penetrate (enter) your partner? (*n* = 94)	
Did not attempt intercourse	35 (37)
Almost never or never	5 (5.3)
A few times (less than half the time)	3 (3.2)
Sometimes (about half the time)	8 (8.5)
Most times (more than half the time)	6 (6.4)
Almost always or always	37 (39)

4. During sexual intercourse, how often were you able to maintain your erection after you had penetrated (entered) your partner? (*n* = 95)	
Did not attempt intercourse	35 (37)
Almost never or never	7 (7.4)
A few times (less than half the time)	4 (4.2)
Sometimes (about half the time)	6 (6.3)
Most times (more than half the time)	10 (11)
Almost always or always	33 (35)
5. During sexual intercourse, how difficult was it to maintain your erection to completion of intercourse? (*n* = 95)	
Did not attempt intercourse	34 (36)
Extremely difficult	6 (6.3)
Very difficult	4 (4.2)
Difficult	4 (4.2)
Slightly difficult	14 (15)
Not difficult	33 (35)
6. How do you rate your confidence that you could get and keep an erection? (*n* = 94)	
Very low	23 (24)
Low	6 (6.4)
Moderate	16 (17)
High	13 (14)
Very high	36 (38)

Percentages may not add up to 100%, as they are rounded.
